# Mental, neurological, and substance use problems among refugees in primary health care: analysis of the Health Information System in 90 refugee camps

**DOI:** 10.1186/s12916-014-0228-9

**Published:** 2014-11-24

**Authors:** Jeremy C Kane, Peter Ventevogel, Paul Spiegel, Judith K Bass, Mark van Ommeren, Wietse A Tol

**Affiliations:** Department of Mental Health, Johns Hopkins Bloomberg School of Public Health, 624 North Broadway, Room 894, Baltimore, MD 21205 USA; Division of Programme Support and Management, United Nations High Commissioner for Refugees, Geneva, Switzerland; Department of Mental Health and Substance Abuse, World Health Organization, Geneva, Switzerland

**Keywords:** Refugee, PTSD, Epilepsy, Global mental health, Service utilization, Trauma, Help-seeking behavior

## Abstract

**Background:**

Population-based epidemiological research has established that refugees in low- and middle-income countries (LMIC) are at increased risk for a range of mental, neurological and substance use (MNS) problems. Improved knowledge of rates for MNS problems that are treated in refugee camp primary care settings is needed to identify service gaps and inform resource allocation. This study estimates contact coverage of MNS services in refugee camps by presenting rates of visits to camp primary care centers for treatment of MNS problems utilizing surveillance data from the Health Information System (HIS) of the United Nations High Commissioner for Refugees.

**Methods:**

Data were collected between January 2009 and March 2013 from 90 refugee camps across 15 LMIC. Visits to primary care settings were recorded for seven MNS categories: epilepsy/seizure; alcohol/substance use; mental retardation/intellectual disability; psychotic disorder; emotional disorder; medically unexplained somatic complaint; and other psychological complaint. The proportion of MNS visits attributable to each of the seven categories is presented by country, sex and age group. The data were combined with camp population data to generate rates of MNS visits per 1,000 persons per month, an estimate of contact coverage.

**Results:**

Rates of visits for MNS problems ranged widely across countries, from 0.24 per 1,000 persons per month in Zambia to 23.69 in Liberia. Rates of visits for epilepsy were higher than any of the other MNS categories in nine of fifteen countries. The largest proportion of MNS visits overall was attributable to epilepsy/seizure (46.91% male/35.13% female) and psychotic disorders (25.88% male/19.98% female). Among children under five, epilepsy/seizure (82.74% male/82.29% female) also accounted for the largest proportion of MNS visits.

**Conclusions:**

Refugee health systems must be prepared to manage severe neuropsychiatric disorders in addition to mental conditions associated with stress. Relatively low rates of emotional and substance use visits in primary care, compared to high prevalence of such conditions in epidemiological studies suggest that many MNS problems remain unattended by refugee health services. Wide disparity in rates across countries warrants additional investigation into help seeking behaviors of refugees and the capacity of health systems to correctly identify and manage diverse MNS problems.

## Background

Studies have repeatedly shown that refugees experience a range of adversity that puts them at risk for mental health problems such as psychological distress, major depression, anxiety disorders and posttraumatic stress disorder (PTSD) [1-6]. Much of the epidemiological literature concerning refugees has focused primarily on the relation between exposure to circumscribed violent events, PTSD and depression [7]. A systematic review of mental health outcomes among populations affected by trauma and displacement revealed substantial variability in rates of common mental disorders across studies, with prevalence rates for PTSD ranging from 0 to 99% and for depression ranging from 3% to 85.5% [5]. Meta-analysis of the most robust of these surveys (that is, those using random samples and diagnostic interviews) showed average prevalence estimates of 15.4% (30 studies) for PTSD and 17.3% (26 studies) for depression [5]. These estimates are substantial given the large number of refugees worldwide. As of June 2013, there were an estimated 38.7 million forcibly displaced people globally, including more than 11 million refugees under the mandate of the United Nations High Commissioner for Refugees (UNHCR) [8]; this includes approximately 6.4 million refugees who had protracted length of stays in refugee camps of five years or more [9].

In addition to these common mental disorders, other types of mental health problems warrant attention in such settings, including a wider range of (often pre-existing) mental, neurological and substance use (MNS) problems, such as psychotic disorders, epilepsy, and alcohol or substance use disorders [6]. Psychotic disorders are associated with high degrees of disability in humanitarian settings [10]; however, little is known about the incidence or prevalence of psychosis among refugees in camps or the burden these types of mental disorders place on health systems within the camps [10,11]. One population-based study reported a 3.3% lifetime prevalence of psychosis among refugees in a camp in Lebanon [12], which is considerably higher than in most other populations [13].

Similarly, few refugee studies have included measures of epilepsy even though it is two to three times more prevalent in sub-Saharan Africa than in high-income countries [14], and refugee populations may be at increased risk for epilepsy because of a higher preponderance of brain injury, infection and poor nutrition [15,16]. Consultations for epilepsy at a clinic in the Kakuma refugee camp in Kenya increased by 70% over a three-year period and represented 16% of all MNS consultations. A recent study of 127 refugee camps found that between 2008 and 2011 there were more than 53,000 health visits due to epilepsy in just over 1,400 camp-months [17].

The epidemiological literature on alcohol and other substance use problems in refugee camps is also limited [18,19]. In one of the few studies on alcohol use among refugees in a camp setting, Luitel *et al*. [20] found that among those who reported drinking any alcohol in a camp in Nepal, the proportion who drank at problematic levels was high and similar to that seen among populations in high-income countries despite a cultural taboo on alcohol use in the camp [20]. Alcohol and substance use were also key concerns among other displaced populations in Africa and Asia [21,22].

Improved knowledge on the range and prevalence of MNS problems being treated at primary care settings in refugee camps is important because this would assist policy decisions regarding resource allocation and required capacity building. There are currently no studies that have estimated contact coverage, or the proportion of the population in need of services that is currently in contact with those services, in low- and middle-income countries (LMIC) [23]. Given the limited resources for mental health in LMIC [24], most primary care clinics in refugee settings have to make difficult choices as to which capacities to prioritize. Data on service utilization may reveal: 1) large gaps between prevalence of MNS problems (for example, as identified in needs assessments or epidemiological studies) and the number and type of MNS problems that are treated in refugee camp primary care settings, prompting the need for investigation into ways to address a possible treatment gap; and/or 2) the need for increased quality control and capacity building to treat certain types of MNS problems for which the data indicate there are high rates of service use.

From a health systems perspective, it is important for UNHCR and its partners to have knowledge of the types of MNS problems that are treated within primary care settings. In January 2009, UNHCR began including MNS indicators in its Health Information System (HIS; subsequently called Twine-- [25]) for primary care settings within refugee camps. The development of the HIS [26] and the rationale for its use have been described elsewhere [27]. Since its inception, HIS data have been used to evaluate UNHCR nutrition program performance in camp settings [28], compare outpatient service utilization between refugee and host communities [29], and estimate incidence and risk factors for malaria, pneumonia, and diarrheal diseases in children under five [30]. This study presents the first examination of data from the HIS MNS indicators, currently collected from 90 refugee camps across 15 countries.

## Methods

### Participant and study setting

This study is a secondary analysis of all MNS data collected through the HIS from January 2009 to March 2013 from 90 refugee camps in 15 participating countries. These 15 countries represent seven UNHCR regions: 1) Central Africa and the Great Lakes (Burundi, Rwanda, Tanzania); 2) East and Horn of Africa (Chad, Djibouti, Ethiopia, Kenya, Uganda); 3) Southern Africa (Namibia, Zambia); 4) West Africa (Liberia); 5) Middle East (Yemen); 6) South Asia (Nepal); and 7) South-East Asia (Bangladesh, Thailand).

### Measures and data collection

A comprehensive HIS reference manual was used as the primary source document in ‘training the trainers’ for each country that implemented HIS [26]. Training of trainers (country-level staff in partner implementing organizations) was conducted over a five-day workshop and the reference manual was used across sites to increase reliability of the training. UNHCR and its partners subsequently trained clinical staff in each refugee camp before initiation of the HIS system.

Data were collected within each refugee camp at the clinic level in outpatient departments and entered on tally sheets by the consulting physician or clinical officer. Seven MNS categories were developed in a consultative process with key experts from the World Health Organization, other United Nations agencies and international non-governmental organizations active in mental health service delivery in humanitarian emergencies. The seven categories present a modification of the categories proposed in the Inter-Agency Standing Committee Guidelines on Mental Health and Psychosocial Support in Emergency Settings [31]. The seven categories comprised: 1) epilepsy/seizures; 2) alcohol/substance use disorder; 3) mental retardation/intellectual disability; 4) psychotic disorder; 5) severe emotional disorder; 6) medically unexplained somatic complaint; and 7) other psychological complaint. Case definitions for each category were developed to suggest probable diagnosis in a primary health care setting (Table 1). These categories were designed to include MNS problems relevant to primary care in refugee settings, keeping in mind that a limited number of categories would enable feasible implementation. Among the consulted experts there was consensus to make a distinction between ‘*severe emotional disorders*’ (such as severe and disabling forms of depression and PTSD) that need to be prioritized within the health care system and ‘*other psychological complaints*’ that would include less severe forms of depressive and anxiety disorders, as well as strong emotional reactions, such as acute stress reactions and grief reactions that are common in emergency settings and that may or may not be pathological.

**Table 1:** **Case definition for mental, neurological and substance use disorders in the UNHCR HIS data**

**Mental, neurological and substance use category**	**Case definition**
Epilepsy/seizures	At least two episodes of seizures not provoked by a cause such as fever, infection, injury or alcohol withdrawal and characterized by loss of consciousness, shaking of limbs, and sometimes with physical injury, incontinence and biting of the tongue.
Alcohol/substance use disorder	Consumption of alcohol or another substance on a daily basis with difficulty controlling consumption. Social relationships, functioning and physical health may deteriorate but use continues.
Mental retardation/intellectual disability	Low intelligence causing problems with functioning. Independent living is rare. In severe cases, a person may have difficulty speaking and understanding others.
Psychotic disorder	Having hallucinations or delusions with disorganized speech or thoughts. People may neglect themselves but may also go through periods of extreme happiness, irritability, talkativeness and recklessness. A person’s behavior is often considered ‘highly bizarre’ by others in the same culture.
Severe emotional disorder	Functioning is significantly impaired for two weeks or more due to 1) overwhelming sadness/apathy; 2) uncontrollable anxiety or fear. Personal relationships, appetite and sleep may be affected. The person may also have suicidal thoughts.
Medically unexplained somatic complaint	Any somatic/physical complaint that does not appear to have an organic cause.
Other psychological complaint (not otherwise specified)	This category covers complaints related to emotions, thoughts or behaviors but does not meet criteria for any of the above categories. Functioning is not impaired or is only moderately impaired. Complaints in this category may be for less severe emotional disorders or normal distress not associated with a disorder.

The data collection tool stratified cases by sex and age group (years). The tool did not allow for distinguishing new and revisit cases.

### Analysis

Data from all 90 refugee camps were combined and then stratified by country, sex, and age group (children younger than five and everyone else five years old and older). The proportion of MNS problems attributable to each of the seven categories is presented.

We also estimated contact coverage of MNS services for each camp by calculating rates of MNS visits per thousand persons per month for each of the seven categories as well as for an overall MNS visit rate. As described by De Silva *et al*., contact coverage refers to the proportion of the population in need of services that is actually in contact with those services [23]. Under ideal circumstances this calculation is made by dividing those receiving services by the estimated number of those in the population who require those services. In the case of refugee camps, however, and indeed most settings in LMIC, the estimated number of those requiring services is unknown or a very rough estimate [32]. Therefore, we have followed the recommendation by De Silva *et al*. and constructed a rate of those accessing services relative to the total camp population size, a measure that has substantial utility in health services planning [23].

Specifically, the rates for our study were calculated by dividing the specific category number of visits, or total number of MNS visits, recorded in the camp during the HIS reporting period by the amount of person-time contributed by the camp, calculated as the average monthly population in the camp during the reporting period multiplied by the number of months HIS was active during that period. The ensuing rate was multiplied by 1,000 to yield MNS visits/1,000/month for the camp. Weighted mean rates and standard deviations were calculated at the country level, as well as across all camps for sex and age categories. Weights were calculated as the ratio of a camp’s contributed person-time to all camps’ contributed person-time within a country (for the country-level means) and the ratio of a camp’s contributed person-time to all camps’ contributed person-time for all 90 camps (for the sex and age category calculations). Population estimates utilized for the rate calculations were extracted from a separate HIS camp population database.

### Ethics

The current analysis was conducted from completely de-identified data. The study was given exempt status by the Institutional Review Board of the Johns Hopkins Bloomberg School of Public Health.

## Results

A monthly average of 1,868,959 million refugees were living in the 90 participating camps between January 2009 and March 2013. During that time period, the total number of reported visits for any MNS disorder complaint was 211,728 resulting in a weighted mean rate across all camps of 4.28 visits per 1,000 persons per month (SD: 4.62). Overall, 40.6% of all MNS visits were attributable to epilepsy/seizures, the highest proportion of all seven categories, followed by psychotic disorders (22.7%), then emotional disorders (12.8%). The smallest proportion of MNS visits was attributable to alcohol/substance use (1.1%) followed by mental retardation/intellectual disability (2.7%).

Table 2 displays the number of camps where HIS was active within each country, the number of calendar months HIS was active in each country, the average monthly refugee population size within each country, and the weighted mean rates of MNS visits per 1,000 refugees per month for each country and MNS category. Countries with the highest mean rates (per thousand per month) of total reported MNS visits (new and revisit) were Liberia (mean: 23.69; SD: 14.56), Nepal (mean: 15.77; SD: 8.74), Burundi (mean: 12.72; SD: 1.52), and Tanzania (mean: 9.99; SD: 1.37). Countries with the lowest rates included Zambia (mean: 0.24; SD: 0.12), Bangladesh (mean: 0.28; SD: 0.33) and Ethiopia (mean: 1.54; SD: 1.66). For nine countries (Burundi, Rwanda, Tanzania, Chad, Djibouti, Kenya, Uganda, Zambia and Thailand) epilepsy/seizure had the highest rate of all MNS categories. In Ethiopia, Namibia, and Nepal psychotic disorders had the highest rate; in Liberia and Yemen severe emotional disorders were highest, and in Bangladesh other psychological complaint had the highest rate.

**Table 2:** **Weighted mean rates of MNS visits per 1,000 refugees per month for each participating HIS country from January 2009 - March 2013**

			**Epilepsy/seizure**	**Alcohol/substance**	**Mental retardation**	**Psychotic disorder**	**Emotional disorder**	**Somatic complaint**	**Other psychological**	**Total**
**Country (number of camps)**	**Number of calendar months HIS was active in country (camp range)** ^**a**^	**Average population per month (camp range)** ^**b**^	**Weighted mean visit rate per thousand per month (weighted SD)** ^**c**^							
			**(number of MNS visits/** **row%)**							
Liberia (3)	6 (2 to 3)	23,420 (5,700 to 9,183)	3.71 (1.33)	1.46 (1.24)	1.44 (0.91)	2.40 (1.55)	7.05 (4.41)	1.99 (2.38)	5.64 (3.70)	23.69 (14.56)
			(229/**15.7**)	(90/**6.2**)	(89/**6.1**)	(148/**10.1**)	(435/**29.8**)	(123/**8.4**)	(348/**23.8**)	(1,462/**100**)
Nepal (7)	43 (19 to 43)	109,692 (5,605 to 33,968)	3.51 (2.20)	0.13 (0.11)	0.11 (0.10)	3.62 (1.32)	1.90 (2.99)	3.48 (2.51)	3.01 (1.37)	15.77 (8.74)
			(9,966/**22.3**)	(369/**0.8**)	(316/**0.7**)	(10,288/**23.0**)	(5,400/**12.1**)	(9,864/**22.0**)	(8,549/**19.1**)	(44,752/**100**)
Burundi (3)	15 (15 to 15)	24,682 (6,535 to 9,869)	5.52 (0.62)	0.09 (0.06)	1.03 (0.73)	2.94 (0.90)	0.79 (0.83)	0.64 (0.52)	1.70 (1.12)	12.72 (1.52)
			(2,043/**43.4**)	(35/**0.7**)	(383/**8.1**)	(1,090/**23.2**)	(292/**6.2**)	(237/**5.0**)	(629/**13.4**)	(4,709/**100**)
Tanzania (2)	35 (35 to 35)	99,484 (37,025 to 62,460)	7.71 (1.75)	0.75 (0.73)	0.12 (0.03)	1.22 (0.35)	0.67 (0.29)	0.19 (0.19)	0.05 (0.04)	9.99 (1.37)
			(26,858/**77.2**)	(75/**0.2**)	(408/**1.2**)	(4,253/**12.2**)	(2,343/**6.7**)	(676/**1.9**)	(188/**0.5**)	(34,801/**100**)
Djibouti (1)	41	14,461	1.92	0.02	0.13	0.43	0.48	1.53	0.47	4.99
			(1,141/**38.6**)	(14/**0.5**)	(80/**2.7**)	(255/**8.6**)	(283/**9.6**)	(908/**30.7**)	(278/**9.4**)	(2,959/**100**)
Yemen (3)	43 (40 to 1)	59,630 (13,876 to 27,025)	0.72 (0.34)	0.03 (0.02)	0.08 (0.03)	0.87 (0.29)	1.47 (0.53)	0.50 (0.42)	0.94 (0.87)	4.61 (2.07)
			(1,750/**15.6**)	(63/**0.6**)	(195/**1.7**)	(2,104/**18.8**)	(3,582/**31.9**)	(1,226/**10.9**)	(2,295/**20.5**)	(11,215/**100**)
Kenya (6)	45 (12 to 45)	513,306 (13,674 to 115,809	1.39 (1.06)	0.05 (0.03)	0.12 (0.06)	0.95 (0.31)	0.63 (0.72)	0.21 (0.17)	0.34 (0.29)	3.71 (2.36)
			(23,846/**37.5**)	(932 / **1.5**)	(2,104/**3.3**)	(16,249/**25.6**)	(10,853/**17.1**)	(3,665/**5.8**)	(5,878/**9.3**)	(63,527/**100**)
Rwanda (3)	37 (27 to 30)	50,728 (13,570 to 18,747)	1.43 (0.95)	0.007 (0.007)	0.07 (0.03)	0.81 (0.65)	0.48 (0.42)	0.19 (0.11)	0.52 (0.47)	3.50 (2.63)
			(1,723/**40.8**)	(8/**0.2**)	(78/**1.9**)	(981/**23.2**)	(576/**13.6**)	(234 / **5.5**)	(625/**14.8**)	(4,225/**100**)
Thailand (9)	51 (45 to 50)	143,839 (3,731 to 44,466)	1.17 (0.56)	0.04 (0.06)	0.07 (0.15)	1.08 (0.61)	0.07 (0.11)	0.11 (0.15)	0.21 (0.22)	2.74 (0.77)
			(7,662/**42.6**)	(248/**1.4**)	(447/**2.5**)	(7,106/**39.5**)	(452/**2.5**)	(697/**3.9**)	(1,392/**7.7**)	(18,004/**100**)
Namibia (1)	29	6,642	0.93	0.11	0.36	0.99	0.0	0.005	0.0	2.39
			(180/**39.1**)	(21/**4.6**)	(69/**15.0**)	(190/**41.2**)	(0/**0.0**)	(1/**0.2**)	(0/**0.0**)	(461/**100**)
Chad (23)	42 (1 to 28)	371,835 (2,323 to 32,252)	1.12 (0.64)	0.06 (0.11)	0.17 (0.13)	0.46 (0.31)	0.31 (0.29)	0.10 (0.30)	0.20 (0.32)	2.38 (1.38)
			(5,614/**47.0**)	(319/**2.7**)	(861/**7.2**)	(2,328/**19.5**)	(1,533/**12.8**)	(524/**6.5**)	(772/**4.4**)	(11,951/**100**)
Uganda (9)	43 (7 to 38)	140,447 (3,875 to 57,310)	0.83 (0.99)	0.03 (0.05)	0.05 (0.05)	0.36 (0.72)	0.23 (0.26)	0.53 (0.33)	0.23 (0.24)	2.25 (2.29)
			(3,016/**36.8**)	(99/**1.2**)	(184/**2.3**)	(1,303/**15.9**)	(849/**10.4**)	(1,914/**23.4**)	(825/**10.1**)	(8,190/**100**)
Ethiopia (12)	41 (2 to 33)	229,672 (5,850 to 39,559)	0.53 (0.59)	0.03 (0.04)	0.12 (0.23)	0.54 (0.87)	0.13 (0.12)	0.10 (0.10)	0.09 (0.13)	1.54 (1.66)
			(1,636/**34.7**)	(101/**2.1**)	(374/**7.9**)	(1,641/**34.8**)	(385/**8.1**)	(308/**6.5**)	(265/**5.6**)	(4,710/**100**)
Bangladesh (3)	46 (30 to 46)	43,272 (11,559 to 17,583)	0.05 (0.05)	0.0006 (0.001)	0.05 (0.09)	0.05 (0.04)	0.05 (0.08)	0.01 (0.02)	0.06 (0.07)	0.28 (0.33)
			(96/**19.2**)	(1/**0.2**)	(95/**19.0**)	(88/**17.6**)	(88/**17.6**)	(21/**4.2**)	(112/**22.4**)	(501/**100**)
Zambia (4)	36 (14 to 35)	37,847 (4,818 to 16,480)	0.14 (0.13)	0.02 (0.02)	0.002 (0.002)	0.05 (0.02)	0.002 (0.003)	0.01 (0.01)	0.005 (0.007)	0.24 (0.12)
			(154/**59.0**)	(25/**9.6**)	(2/**0.8**)	(59/**22.6**)	(2/**0.8**)	(14/**5.4**)	(5/**1.9**)	(261/**100**)

^a^Refers to the number of calendar months HIS was active in the country. HIS was not always active at the same time in each camp within a country. There were often periods of overlap but also months in which HIS was active in some camps but not others. The lower end of the range refers to the camp within the country that had the smallest number of calendar months and the upper number the camp with the highest number of calendar months. There are no ranges provided for countries with only one camp. Data displayed in this column are for reference purposes and were not explicitly used to calculate the rates in the subsequent columns. These were calculated at the camp level not the country level (see footnote *c* below). ^b^Average population per month for each country calculated as the sum of the average monthly population for each camp within the country. The lower end of the range refers to the camp within the country with the smallest average monthly population size and the upper number the camp with the highest average monthly population size. ^c^Rates were first calculated at the camp level. For each camp, the numerator of the rate was the total number of MNS visits recorded during the months HIS was active in that camp. The denominator was person-time, calculated as the average monthly population in the camp multiplied by the number of months HIS was active in the camp. The resulting rate was multiplied by 1,000. For each country, a weighted mean rate (and standard deviation) was calculated from the camp rates within that country. Weights were calculated as the ratio of a camp’s contributed person-time (average monthly population multiplied by the number of months HIS was active) to all contributed person-time in the country. Therefore the weights summed to 1. HIS, Health Information System; MNS, mental, neurological and substance use.

### Sex

Table 3 summarizes the mean MNS visit rates separately for males and females. Overall, females (mean: 4.57; SD: 5.70) had a higher average MNS visit rate per 1,000 per month compared to males (mean: 4.00; SD: 3.77). Females also had higher rates of emotional disorder, medically unexplained somatic complaint and other psychological complaint visits compared to males who, in turn, had higher rates than females of epilepsy/seizure, alcohol/substance use, mental retardation and psychotic disorder visits. Despite these differences, however, for both males (46.9%) and females (35.1%), epilepsy/seizure accounted for the greatest percentage of MNS visits recorded and alcohol/substance use accounted for the smallest percentage (2.0% among males and 0.4% among females).

**Table 3:** **Weighted mean rates of MNS visits per 1,000 refugees per month by sex and age group from January 2009 to March 2013**

	**Male**			**Female**		
	**<5 years old**	**5 and above**	**Total**	**<5 years old**	**5 and above**	**Total**
**MNS Category**	**Weighted mean visit rate per thousand per month (weighted SD)** ^**a**^					
	**(number of MNS visits/** **column%)**					
Epilepsy/seizure	0.93 (1.54)	2.36 (3.52)	1.88 (2.17)	0.66 (1.05)	2.29 (3.69)	1.61 (2.10)
	(3,854/**82.7**)	(42,051/**45.1**)	(45,905/**46.9**)	(2,700/**82.3**)	(37,309/**33.7**)	(40,009/**35.1**)
Alcohol/substance	0.002 (0.01)	0.10 (0.19)	0.08 (0.15)	0.0001 (0.01)	0.02 (0.06)	0.02 (0.05)
	(9/**0.2**)	(1,965/**2.1**)	(1,974/**2.0**)	(4/**0.1**)	(422/**0.4**)	(426/**0.4**)
Mental retardation	0.12 (0.27)	0.15 (0.23)	0.13 (0.17)	0.07 (0.16)	0.19 (0.55)	0.10 (0.17)
	(493/**10.6**)	(2,664/**2.9**)	(3,157/**3.2**)	(295/**9.0**)	(2,233/**2.0**)	(2,528/**2.2**)
Psychotic disorder	0.01 (0.02)	1.32 (1.21)	1.03 (0.92)	0.01 (0.03)	1.38 (2.22)	0.91 (1.06)
	(35/**0.8**)	(25,291/**27.1**)	(25,326/**25.9**)	(55/**1.7**)	(22,702/**20.5**)	(22,757/**20.0**)
Emotional disorder	0.02 (0.08)	0.54 (1.14)	0.39 (0.81)	0.01 (0.04)	1.06 (2.07)	0.70 (1.25)
	(70/**1.5**)	(9,592/**10.3**)	(9,662/**9.9**)	(47/**1.4**)	(17,364/**15.7**)	(17,411/**15.3**)
Somatic complaint	0.01 (0.09)	0.48 (2.48)	0.22 (0.48)	0.01 (0.13)	0.77 (1.83)	0.60 (1.59)
	(40/**0.9**)	(5,399/**5.8**)	(5,439/**5.6**)	(61/**1.9**)	(14,912/**13.5**)	(14,973/**13.2**)
Other psychological	0.04 (0.10)	0.37 (0.79)	0.26 (0.49)	0.03 (0.06)	0.80 (1.41)	0.63 (1.23)
	(157/**3.4**)	(6,228 / **6.7**)	(6,385/**6.5**)	(119/**3.6)**	(15,657/**14.2**)	(15,776/**13.9**)
**Total**	1.13 (1.75)	5.32 (7.86)	4.00 (3.77)	0.80 (1.20)	6.52 (9.56)	4.57 (5.70)
	(4,658/**100**)	(93,190/**100**)	(97,848/**100**)	(3,281/**100**)	(110,599/**100**)	(113,880/**100**)

^a^Rates were first calculated at the camp level. For each camp, the numerator of the rate was the total number of MNS visits recorded during the months HIS was active in that camp for the specific sex/age category. The denominator was person-time, calculated as the average monthly population in the camp multiplied by the number of months HIS was active in the camp for each specific age/sex category. The resulting rate was multiplied by 1,000. A weighted mean rate (and standard deviation) was calculated from all camp rates within each sex/age category. Weights were calculated as the ratio of a camp’s contributed person-time (average monthly population multiplied by the number of months HIS was active) to all contributed person-time from all camps. Therefore the weights summed to 1. HIS, Health Informatin System; MNS, mental, neurological and substance use.

### Children younger than five

MNS visit rates per 1,000 were lower among children younger than five years compared to those five and older for all categories (Table 3). Overall, MNS rates were higher among males younger than five (mean: 1.13; SD: 1.75) compared to females younger than five (mean: 0.80; SD: 1.20). For both male (82.7%) and female (82.3%) children younger than five, epilepsy/seizure accounted for the greatest proportion of MNS visits, followed by mental retardation (males: 10.6%; females: 9.0%) and other psychological complaint (males: 3.4%; females: 3.6%). Rates of alcohol/substance use, psychotic disorder, emotional disorder, medically unexplained somatic complaint and other psychological complaint were all very low for both sexes, with no rates greater than 0.10 per 1,000 per month.

## Discussion

This is the largest study of MNS problems among refugees visiting primary care in camps to date. The data have revealed several important findings. First, the HIS data indicated substantial differences in contact coverage across countries, as measured by MNS visit rates. In theory, it is possible that these disparities are due to actual differences in population rates of these disorders, perhaps due to differential risk factors across regions or refugee camp settings. However, more likely explanations include variation across sites in the degree of training or ability to recognize MNS among medical practitioners, differences in accessibility of the medical services within the refugee camps, or differences in the quality of treatment and availability of medication. These possible explanations and ways to address them are discussed in more detail below.

Our second result is the marked gender differences among the various MNS problems. Our data indicate much higher rates of alcohol/substance use visits among males as compared to females, confirming findings from studies in general populations in high-, middle- and low-income countries [33] and specifically among forcibly displaced populations [18,0]. Moreover, the literature indicates that women are often less likely to seek services for alcohol and substance use problems than men [34].

Similar to findings from previous studies with refugee populations [15,35,36], the rate of emotional disorder visits was substantially higher among females on average (0.70 visits/1,000 females/month) compared to males (0.39 visits/1,000 males/month) while males had slightly higher rates of psychotic disorder visits (1.03) than females (0.91). Rates for medically unexplained somatic complaint and other psychological complaint were more than twice as high among females as among males. The higher medically unexplained somatic complaint rates among females are also consistent with the epidemiological literature [37,38]. The higher rates for women of medically unexplained somatic complaints as well as other psychological complaints may lead to poor matching of health interventions with underlying conditions, thus indicating the need for detailed training in mental health for personnel in primary care refugee settings.

Our third major finding concerns MNS visit rates among children younger than five years old. The most common MNS visit among this group was for epilepsy/seizure, with a higher rate among males (0.93) as compared to females (0.66). Although absolute rates of epilepsy visits differed between males and females younger than five years old, epilepsy/seizure accounted for an almost identical proportion of all MNS visits for males (82.7%) as for females (82.3%) given that males had higher overall visit rates. Rates of other MNS visits for children younger than five years were extremely low, at less than 0.15 per 1,000 per month for both males and females.

Our fourth key finding is the higher rate of epilepsy and psychotic disorder visits relative to the other MNS categories. Across the majority of countries and among both males and females we found greater contact coverage for epilepsy and psychotic disorders compared to other MNS problems. Previous research has suggested that rates of service use for severe mental and neurological disorders may be higher than service use for other MNS problems in humanitarian settings [12,39,40] and that refugee populations, in particular, may be at an increased risk for psychotic disorders, epilepsy and other neuropsychiatric conditions [15,36]. From this perspective, our finding that epilepsy/seizures (40.6%) and psychotic disorders (22.7%) were responsible for the highest proportion of MNS disorder visits overall and in a majority of the 15 countries is important given the scant attention these disorders have received in population based epidemiological studies.

Conversely, the fifth major finding is that service use for emotional disorders, such as depression, anxiety disorders and PTSD, is low relative to psychotic disorders and epilepsy as well as in absolute terms. The health care system in the refugee camps seems not to cater adequately for people with emotional disorders.

**Figure 1:**
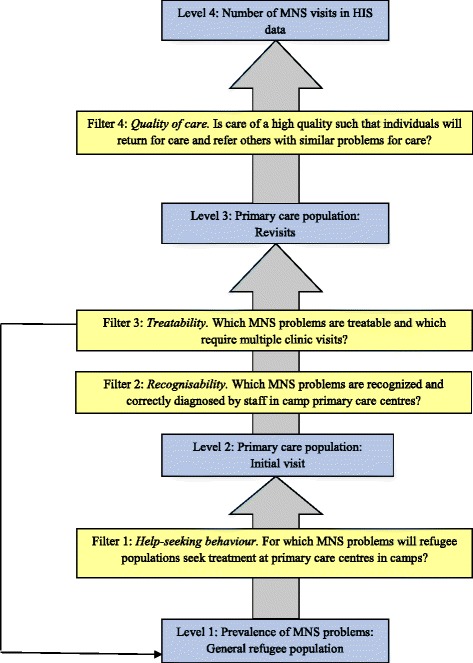
**Conceptual framework of MNS visits in HIS data.** HIS, Health Information System; MNS, mental, neurological and substance use.

To help explain our findings, and to identify gaps in knowledge, we use a conceptual framework adapted from the classic model by Goldberg and Huxley [41]. The original framework was offered to conceptualize pathways to mental health care through five levels separated by four filters. We have adapted this framework for refugee settings (Figure 1).

Help-seeking behavior (Filter 1) may vary widely for different types of MNS problems. Refugees, as with other populations in LMIC, are likely to first seek services for mental health care within their families and with traditional and religious healers [42,43], and if these services are perceived as helpful will not seek services from health clinics. An important determinant of help-seeking behavior is related to the interpretation of symptoms by the refugees themselves. For example, community members in four conflict-affected settings in Africa described syndromes that were similar in many ways to disorders described in psychiatric classification systems (for example, psychotic and mood disorders). Syndromes that resembled psychotic disorders were viewed as very serious medical problems that required treatment while syndromes akin to emotional disorders were not seen as medical disorders and were expected to resolve primarily through social support mechanisms or religious and traditional healing [44]. Similar patterns have been observed elsewhere in African [45-48] and Asian settings [49].

It is likely that there are major differences in the accuracy (Filter 2) with which MNS problems will be recognized and correctly diagnosed by staff in camp primary care centers. For example, medical staff in primary care settings may be trained in the identification of severe mental disorders, but not in those of other disorders [49]. Without appropriate training of clinical staff and integration of mental health services into primary care, many people suffering from emotional disorders may be misdiagnosed and treated for somatic symptoms or physical illness [50-52].

MNS problems vary considerably in the degree to which they can be successfully treated (Filter 3) and in the type and level of skill required of primary health care staff. Treatment of an individual with psychosis, for example, calls for a variety of skills including communication techniques and an aptitude to work closely with the individual and the support system around him or her. Moreover, chronic and difficult to treat problems may require more health visits.

The level of service user satisfaction may largely determine whether users return for care (Filter 4) and refer others with similar problems for care. If regular supply of medication is guaranteed to mitigate symptoms, it is more likely that users will return, particularly for chronic disorders that involve long-term treatment with drugs. Studies conducted in humanitarian settings indicate that epilepsy and psychosis have high rates of return visits while emotional disorders have much lower rates of return visits [15,40,53].

### Limitations

There are several important limitations of our study to consider. First, the HIS data collection forms for MNS problems did not differentiate between new and revisits making the measurement of incidence rates impossible and limiting our ability to make comparisons to the epidemiological literature or expected rates of disorders among this population. Second, the HIS categories were made for use in general health systems in refugee settings and are not fully compatible and comparable with data based on established classification systems, such as the Diagnostic and Statistical Manual of Mental Disorders, Fourth Edition (DSM-IV) and the 10 revision of the International Classification of Diseases (ICD-10). Third, the HIS does not elucidate comorbidity, a significant limitation given the expected high rates of co-occurring MNS problems. Fourth, there was wide variation in the number of months each camp collected HIS data. Liberia, for example, only had six calendar months of reporting time. We accounted for this in part, however, by calculating rates of MNS visits per month. Fifth, although the HIS MNS visit data had age group categories of 0 to 4, 5 to 17, 18 to 59, and 60 years and older, the population data available to calculate the rates only differentiated between younger than five years old and those older than five years, limiting our ability to analyze differences by these more specific age group categories. Sixth, we had no measure of MNS problem severity, only that the problem was deemed severe enough to warrant a visit to a health clinic. Finally, although training of staff who collect HIS data was uniform, there was undoubtedly variation across countries and across camps in how reliably and accurately data were collected.

## Conclusions

This study is the first attempt to measure contact coverage of MNS services in LMIC. The relatively high number of visits for epilepsy and psychotic disorders reported in the refugee HIS data support calls for more attention to quality care of people with these types of morbidities in refugee settings [11] and for future population-based epidemiological research to include methods to measure their incidence and prevalence. Conversely, there is a major discrepancy between the very low rates of service use for emotional disorders compared to high population rates for PTSD, anxiety and depression reported in the epidemiological literature. There is, therefore, an urgent need to explore how best to develop interventions to serve refugees with these disorders and who currently do not get appropriate treatment. There were striking differences in MNS visit rates by gender and across countries. Differences by gender indicate the need for dedicated training on mental health to match treatments with underlying conditions, when men and women show different patterns in presenting symptoms. The different rates by country may be an indication of disparity in service utilization. Very low rates were found for children younger than five years old.

We presented a public mental health model that helps to explain how rates of disorder at the population level may translate to contact coverage of MNS services at primary health centers. Overall, further efforts are required at all levels of this model to ensure optimal matching of primary health care services with mental health needs and to reduce the treatment gap. This requires a better understanding of ways in which refugees conceptualize distress, their help-seeking behaviors, and matching this with active community outreach, improved recognition of mental health in primary care, and further efforts to implement evidence-based treatments and ensuring increased coverage of effective care for MNS problems with the greatest burden on the lives of refugees.

## Abbreviations

HIS: Health Information System
LMIC: low- and middle-income country
MNS: mental, neurological, and substance use
PTSD: post-traumatic stress disorder
UNHCR: United Nations High Commissioner for Refugees
